# Development, psychometric validation, and correlates of the 15-item quality of life in epilepsy scale (QOLIE-15)

**DOI:** 10.1038/s41598-026-46379-z

**Published:** 2026-03-29

**Authors:** Mariam Dabbous, Fouad Sakr, Pascale Salameh, Pierre-Marie Preux

**Affiliations:** 1 Inserm U1094, IRD UMR270, Univ. Limoges, CHU Limoges, EpiMaCT-Epidemiology of Chronic Diseases in Tropical Zone, Institute of Epidemiology and Global Health-Michel Dumas, OmegaHealth, Limoges, France; 2 School of Pharmacy, Lebanese International University, Beirut, Lebanon; 3 INSPECT-LB: Institut National de Santé Publique, d’Épidémiologie Clinique et de Toxicologie-Liban, Beirut, Lebanon; 4 Faculty of Public Health, Lebanese University, Fanar, Lebanon; 5 Gilbert and Rose-Marie Chagoury School of Medicine, Lebanese American University, Byblos, Lebanon; 6 Department of Primary Care and Population Health, University of Nicosia Medical School, Nicosia, Cyprus

**Keywords:** Epilepsy, Quality of life, QOLIE-15, Validation, Psychometrics, Diseases, Health care, Medical research, Neurology, Neuroscience

## Abstract

Health-related quality of life (HRQoL) is a central outcome in epilepsy care, but existing tools are either lengthy, too brief, or lack sensitivity to epilepsy-specific concerns. This study developed and validated the 15-item Quality of Life in Epilepsy Scale (QOLIE-15) and examined sociodemographic, socioeconomic, clinical, and psychosocial determinants of HRQoL. A cross-sectional study was conducted among adult patients with epilepsy recruited from community pharmacies and a primary healthcare center. Participants completed the QOL assessment alongside established measures of medication adherence, adverse events, cognition, anxiety, and stigma. The dataset was randomly split for exploratory and confirmatory factor analysis (EFA and CFA). Reliability, convergent, concurrent, and criterion validity were assessed. Measurement invariance was tested across gender, seizure characteristics, and seizure control. Multivariable linear regression identified independent predictors of QOLIE-15 scores. The study included 649 patients. EFA supported a stable 5-factor structure, with sampling adequacy confirmed by KMO = 0.875 and Bartlett’s test (*P* < 0.001). CFA demonstrated excellent fit (CFI = 0.978, TLI = 0.972, RMSEA = 0.041, SRMR = 0.034). Internal consistency was high (α = 0.906, ω = 0.929). The QOLIE-15 correlated strongly with QOLIE-31 (*r* = 0.875) and showed excellent criterion validity, with a cut-off of 44.5 yielding sensitivity of 88.8% and specificity of 82.0%. Measurement invariance was established across key subgroups. In multivariable analysis, lower QOL was independently associated with uncontrolled seizures (B = − 1.53), adverse drug effects (B = − 0.10), stigma (B = − 0.38), cognitive complaints (B = − 0.28), anxiety (B = − 0.52), and higher household crowding (B = − 0.77). The QOLIE-15 is a valid, reliable, and practical instrument capturing epilepsy-specific HRQoL. It balances brevity with multidimensionality, and its invariance and robust psychometric properties support application in clinical practice and research. Independent determinants highlight the importance of addressing seizure control, treatment tolerability, stigma, cognition, and anxiety in epilepsy care.

**Supplementary Information:**

The online version contains supplementary material available at 10.1038/s41598-026-46379-z.

## Introduction

Epilepsy is a chronic neurological condition characterized by recurrent seizures and accompanied by a wide spectrum of physical, psychological, and social challenges that affect both patients and their families^1–3^. In recent years, health-related quality of life (HRQoL) has been recognized as a central outcome in epilepsy care, complementing traditional clinical indicators such as seizure frequency or drug efficacy^4^. HRQoL measures are increasingly applied to evaluate the overall wellbeing of patients with epilepsy^5^. Generic HRQoL instruments such as the EQ-5D are widely used in clinical practice and research due to their brevity and standardized structure^6–9^. However, these tools often lack sensitivity to epilepsy-specific concerns, particularly in populations experiencing seizure burden, cognitive impairment, or drug-related side effects^10^. For this reason, disease-specific instruments have been developed to more comprehensively capture the unique aspects of living with epilepsy.

The Quality of Life in Epilepsy Inventory (QOLIE-31) remains the most widely used epilepsy-specific measure^11^. It evaluates seven domains, including seizure worry, emotional wellbeing, cognition, medication effects, and social functioning. Although validated across diverse populations, its length (31 items) and complex scoring system limit practicality in routine use. Patients with cognitive fatigue or limited health literacy may find it burdensome, while clinicians and researchers face challenges in score calculation and interpretation. To address these limitations, the shorter QOLIE-10 was introduced^12^. While easier to administer, the QOLIE-10 provides only a limited overview and may oversimplify the multidimensional impact of epilepsy on HRQoL, reducing its utility for research or detailed clinical assessment. Furthermore, validation studies have reported inconsistencies in the factorial structure and psychometric performance of both QOLIE-31 and QOLIE-10^13–18^. Exploratory factor analyses often failed to replicate the original models, raising concerns about cross-cultural validity and structural robustness. These shortcomings underscore the need for a modern, concise, yet comprehensive multidimensional tool capable of reliably capturing epilepsy-specific HRQoL while remaining practical in diverse settings.

The need for such instruments is especially pressing in Lebanon, a lower-middle-income country in the Middle East facing severe socioeconomic challenges. Medication shortages, financial constraints, and disruptions in healthcare delivery have increased the burden on patients with chronic diseases, including epilepsy^19,20^. Despite this, research on HRQoL in Lebanese patients with epilepsy is scarce, limited in scope, and often conducted without culturally adapted or validated tools. Existing studies also fail to capture the psychosocial and clinical dimensions of living with epilepsy in the current socioeconomic context.

To address these gaps, the present study aims to develop and validate the 15-item Quality of Life in Epilepsy Scale (QOLIE-15), a concise, yet comprehensive, epilepsy-specific, and multidimensional instrument. The scale is designed to capture seizure-related concerns, cognitive functioning, psychological wellbeing, therapeutic side effects, and social functioning. In addition, the study seeks to examine sociodemographic, socioeconomic, clinical, and psychosocial factors associated with HRQoL in patients with epilepsy, in order to provide context-specific insights that may inform clinical practice and healthcare planning.

## Methods

### Study design and participants

A cross-sectional study was conducted among adult patients with epilepsy in Lebanon. Recruitment was carried out through community pharmacies across the country (Beirut, Mount Lebanon, North, South, and Bekaa). Pharmacy medical records were first screened to identify eligible patients, and individuals presenting for antiepileptic drug refills, whether patients themselves or their caregivers, were also screened. Additional participants were recruited from a major primary healthcare center in Beirut that provides specialized care for patients with epilepsy. Inclusion criteria were Lebanese adults (≥ 18 years) with a confirmed epilepsy diagnosis who were receiving at least one antiepileptic medication. Exclusion criteria included non-Lebanese patients, children, individuals not on medication, and those prescribed antiepileptic drugs for non-epileptic indications (e.g., migraine prevention, weight control).

Data were collected through an electronic questionnaire administered by trained interviewers in face-to-face sessions lasting about 20 min, including both the interview and data entry. Each interview started with a short introduction that explained the study objectives and highlighted the potential impact of its findings on patients’ quality of life (QOL). Clinical information was verified with patients’ neurologists or primary care providers when necessary. Prior to full data collection, a pilot study with 20 participants was conducted to assess clarity and comprehensibility. Minor adjustments were made based on feedback, and pilot data were excluded from the final analysis. The main study data were collected between February and August 2025 using a structured Arabic-language questionnaire adapted to the Lebanese context.

### Measures and variables

The study questionnaire was organized into five sections. The first section captured sociodemographic and socioeconomic characteristics, including age, gender, area of residence, marital status, number of children, education, occupation, smoking, alcohol use, access to healthcare, and health coverage. Body Mass Index (BMI) was calculated from weight and height, and the House Crowding Index was computed by dividing household size by the number of rooms. Household monthly income and financial status were also assessed. Financial wellbeing and distress were evaluated using the InCharge Financial Distress/Financial Wellbeing Scale (IFDFW), an 8-item validated instrument scored from 1 to 10, where higher scores reflect greater financial wellbeing^21^. In the present sample, Cronbach’s α = 0.926 and McDonald’s ω = 0.948.

The second section addressed clinical information, including medical history, antiepileptic and concomitant medications, medication coverage by insurance, and barriers to medication access in Lebanon. Seizure characteristics, type, and control were also documented and verified with treating neurologists or primary care providers. Medication adherence was measured using the Lebanese Medication Adherence Scale (LMAS-14), a 14-item validated tool across several chronic conditions^22,23^. Items are rated on a 4-point scale (1 = lower adherence to 4 = higher adherence), with higher total scores indicating better adherence. In this study, Cronbach’s α = 0.964 and McDonald’s ω = 0.980. Adverse antiepileptic drug effects were evaluated using the Liverpool Adverse Events Profile (LAEP)^24,25^, a 19-item tool scored on a 4-point Likert scale (1 = never, 4 = always). The summed score reflects the burden of medication side effects, with higher scores indicating greater adverse events. In this sample, Cronbach’s α = 0.928 and McDonald’s ω = 0.939.

The third section assessed QOL using the Quality of Life in Epilepsy Inventory (QOLIE-31)^11^. Responses were recoded to a 0-100 scale, and weighted formulas were applied to generate a total score, with higher scores reflecting better QOL. In the current sample, Cronbach’s α = 0.915 and McDonald’s ω = 0.932. Stigma was measured with the Epilepsy Stigma Scale (ESS)^26^, a 3-item instrument producing a total score from 0 to 3, where higher values indicate stronger perceived stigma. Cronbach’s α = 0.944 and McDonald’s ω = 0.948 in this study.

The fourth section evaluated cognition using the A-B Neuropsychological Assessment Schedule (ABNAS)^27^. This 24-item self-report tool assesses cognitive difficulties associated with epilepsy and antiepileptic therapy. Items are rated on a 4-point scale (0 = no problem to 3 = severe problem), and the summed score represents overall cognitive impairment. Cronbach’s α = 0.976 and McDonald’s ω = 0.980 in this sample.

The fifth section examined psychological wellbeing using the Lebanese Anxiety Scale (LAS-10)^28^. This validated 10-item tool, culturally adapted to the Lebanese population, screens for anxiety and related symptoms (depression, physical, cognitive, behavioral, and sleep disturbances) during the preceding seven days. Higher total scores correspond to greater anxiety levels. In this study, Cronbach’s α = 0.921 and McDonald’s ω = 0.944.

Formal authorization to use all instruments included in this study was obtained from their respective copyright holders prior to data collection.

### Development of the 15-item quality of life in epilepsy scale (QOLIE-15)

The development of the QOLIE-15 followed established guidelines for patient-reported outcome measures^29^. A comprehensive literature review was conducted to identify existing instruments addressing QOL in epilepsy and its related factors. Items were selected and conceptually integrated to construct a new multidimensional instrument tailored to epilepsy, drawing on relevant content from multiple validated tools rather than constituting a direct short-form adaptation of any single instrument. An initial pool of 28 items (Appendix 1) was generated, comprising 2 items from the LMAS-14^22,23^, 15 from the QOLIE-31^11^, 6 from the ABNAS^27^, and 5 from the LAS-10^28^. This pool was reduced to 18 items (Appendix 2) by removing redundancies and prioritizing comprehensiveness, brevity, and specificity.

A panel of five independent experts in epidemiology, public health, neurology, psychiatry, and epilepsy care evaluated the 18 items for relevance, clarity, and comprehensiveness. Using a 3-point rating scale (1 = least likely to keep, 3 = most likely to keep) and providing qualitative feedback, items were retained if ≥ 80% of experts rated them as “most likely to keep.” The panel also reviewed the initial item pool for transparency.

The final QOLIE-15 consists of 15 items, each retaining the original response format from the source instrument (Appendix 3). The total score is calculated as the sum of all item responses and ranges from 15 to 68, with higher scores indicating better QOL. This straightforward scoring approach was selected to support easy implementation in both clinical and research settings, without requiring complex calculations. Although several items were drawn from the QOLIE-31, the QOLIE-15 is not intended as a formal abbreviated version of that instrument. Instead, it represents a newly constructed multidimensional measure integrating selected items from QOLIE-31 alongside items from ABNAS and LAS-10, reorganized within a unified scoring framework and validated as an independent factorial model. The designation “QOLIE-15” reflects the instrument’s focus on quality of life in epilepsy and its 15-item structure, consistent with common psychometric naming conventions, and is not meant to imply official derivation from previously established QOLIE versions.

To further ensure content validity and patient-centeredness, the preliminary final version was piloted with 20 patients with epilepsy, who evaluated the scale’s clarity, relevance, and comprehensibility. Feedback confirmed the adequacy of the final items.

### Translation procedure

The instruments were translated into Arabic following a forward-backward translation approach by one of the study authors, a Lebanese epidemiologist fluent in English. The resulting version was then back-translated into English. To further validate the accuracy and cultural appropriateness of the translation, both the original and translated versions were reviewed by an independent translator, who addressed and corrected any inconsistencies. Discrepancies in wording or meaning were resolved collaboratively by the authors and the independent translator.

### Ethical aspects

The Ethics and Research Committee of the School of Pharmacy, Lebanese International University, approved the study protocol (Approval number: 2025ERC-011-LIUSOP). Written informed consent was secured from every participant before inclusion. The research adhered to the ethical standards of the Declaration of Helsinki, with confidentiality of participant information maintained throughout the study.

### Sample size calculation

Sample size requirements were determined using two approaches. First, the CDC Epi Info version 7.2.6 population survey tool was applied. In the absence of epilepsy prevalence data in Lebanon, the expected frequency was set at 50% to yield the largest possible minimum sample. This produced a requirement of 384 participants to achieve a 95% confidence level with a 5% margin of error. Second, the minimum sample was estimated using G*Power version 3.1.9.7 (Heinrich Heine Universität Düsseldorf, Germany). A multiple linear regression was planned to examine predictors of the QOLIE-15 score. Assuming a small effect size (f^2^ = 0.0526, corresponding to an R^2^ of 0.05 in the omnibus test), with α = 0.05, power = 0.80, and up to 25 predictors, the minimum required sample size was 454 participants. For the scale validation component, a participant-to-item ratio of 10:1 was adopted^30^, requiring at least 150 individuals for the 15 items of the QOLIE-15. As validation was planned in two independent subsamples, a total of 300 patients was required. Therefore, the final minimum sample size was set at 454, satisfying the requirements for both validation and multivariable regression, ensuring 80% statistical power, 95% confidence level, and an acceptable margin of error of 5%.

### Statistical analysis

All analyses were conducted using R version 4.5.0 (R Foundation for Statistical Computing, Vienna, Austria) with RStudio version 2025.05.0 + 496 (Mariposa Orchid, RStudio, PBC). Descriptive analyses summarized sociodemographic, socioeconomic, clinical, and psychosocial characteristics. Continuous variables are presented as means with standard deviations (± SD), and categorical variables as frequencies and percentages.

The full sample was randomly split in R into two equal samples (Sample 1 and Sample 2). Exploratory factor analysis (EFA) was performed on Sample 1 using the *psych* and *GPArotation* packages, with Promax rotation selected due to expected correlations among the QOLIE-15 items. Suitability was assessed using the Kaiser-Meyer-Olkin (KMO) measure and Bartlett’s test of sphericity. Factors were retained when eigenvalues exceeded 1. Confirmatory factor analysis (CFA) was subsequently conducted on Sample 2 with the *lavaan* package, using maximum likelihood estimation to confirm the structure identified in the EFA. Model fit was assessed using χ^2^/df, Comparative Fit Index (CFI), Tucker-Lewis Index (TLI), Root Mean Square Error of Approximation (RMSEA), and Standardized Root Mean Square Residual (SRMR). Good fit was indicated by χ^2^/df < 3, CFI and TLI ≥ 0.95, RMSEA ≤ 0.06 (≤ 0.08 acceptable), and SRMR ≤ 0.08^31,32^. IBM SPSS Amos version 24.0 (IBM Corp., Armonk, NY, USA) was used to visualize the CFA model and report standardized loadings and factor correlations.

All subsequent analyses were conducted on the total sample. To ensure structural equivalence and valid score comparability across demographic and clinical subgroups, multi-group CFA was performed to test measurement invariance across gender (male vs. female), seizure characteristics (generalized vs. focal), and seizure control (controlled vs. uncontrolled). Configural, metric, and scalar invariance were evaluated, with invariance supported when ΔCFI ≤ 0.010, ΔRMSEA ≤ 0.015, and ΔSRMR ≤ 0.010^33^.

Internal consistency was assessed using Pearson correlations (r) between the QOLIE-15 total score, subscale scores, and individual items. Additional reliability estimates were calculated using polychoric Cronbach’s α and McDonald’s ω, with α obtained via the *psych* package and ω via *semTools*.

Construct validity was examined through convergent and concurrent validity. Convergent validity was evaluated using correlations with QOLIE-31, while concurrent validity was tested via correlations with LMAS-14, LAEP, ABNAS, ESS, and LAS-10. Criterion validity was assessed using receiver operating characteristic (ROC) curve analysis, implemented with the *pROC* and *ggplot2* packages. The QOLIE-31, dichotomized at the median, served as the external criterion, a common practice in the absence of a standard cut-off^34,35^. The optimal QOLIE-15 threshold was identified using Youden’s J index, and the corresponding sensitivity and specificity were reported.

Associations between QOLIE-15 total scores and sociodemographic, socioeconomic, clinical, and psychosocial factors were examined using theory- and practice-driven multivariable linear regression models. Variables were prespecified based on their theoretical, clinical, and practical relevance to epilepsy-related QOL, supported by prior literature and contextual applicability in the Lebanese healthcare setting. Model 1 included sociodemographic and socioeconomic variables (age, House Crowding Index, area of residence, marital status, level of education, occupation, smoking status, alcohol consumption, easy access to healthcare, household monthly income, and IFDFW score). Model 2 included clinical and psychosocial variables (type of seizure, level of seizure control, number of antiepileptic drugs used, LAEP score, LMAS-14 score, medication coverage by a public or private insurance provider, difficulty obtaining medications, need to obtain medications from outside Lebanon, total number of comorbidities, number of daily non-antiepileptic medications, ESS score, ABNAS score, use of other nervous system medications, and LAS-10 score). Model 3 combined significant variables from Models 1 and 2 to produce a parsimonious integrated model. For each model, unstandardized (B) and standardized (β) coefficients, 95% confidence intervals (CI), and P values were reported, with statistical significance set at *P* < 0.05.

## Results

### Sociodemographic and socioeconomic characteristics

This study included 649 patients with epilepsy, with a mean age of 34.84 (± 15.42) years, mean BMI of 25.75 (± 4.98), and mean House Crowding Index of 1.19 (± 0.54). Females slightly outnumbered males (53.16% vs. 46.84%). The largest proportions resided in Bekaa (28.35%) and South Lebanon (27.89%). More than half were single (58.24%), and nearly half (46.07%) holding a university level of education. Unemployment was reported by 52.23% of participants. Most were non-smokers (61.79%), and the vast majority did not consume alcohol (91.53%). Easy healthcare access was reported by 77.04%. The most common payment method was self-payment (43.76%), while household income most frequently ranged between 500 and 1000 USD (39.10%). The mean IFDFW score was 41.14 (± 16.93). Detailed sociodemographic and socioeconomic characteristics are shown in Table 1.

**Table 1:** Sociodemographic and socioeconomic characteristics of the study sample.

Variable	Mean or frequency	SD or %
Age	34.84	15.42
Gender		
Male	304	46.84
Female	345	53.16
BMI	25.75	4.98
House Crowding Index	1.19	0.54
Area of residence		
Beirut	128	19.72
Bekaa	184	28.35
Mount Lebanon	68	10.48
North Lebanon	88	13.56
South Lebanon	181	27.89
Marital status		
Single	378	58.24
Married	241	37.13
Divorced/widowed	30	4.62
Number of children (if any)	1.11	1.69
Level of education		
Not educated	106	16.33
School level	244	37.60
University level	299	46.07
Occupation		
Employed/self-employed	288	44.38
Unemployed	339	52.23
Retired	22	3.39
Smoking status		
Non-smoker	401	61.79
Ex-smoker	24	3.70
Current smoker	224	34.51
Alcohol consumption		
No	594	91.53
In the past, not anymore	29	4.47
Yes, currently	26	4.01
Easy access to healthcare		
No	149	22.96
Yes	500	77.04
Health coverage		
Private insurance	150	23.11
National Social Security Fund (NSSF)	100	15.41
Ministry of Public Health	28	4.31
Public insurance (Army, COOP, Internal Security Forces)	87	13.41
Self-payer	284	43.76
Household monthly income		
Less than 500 USD	219	33.70
500 USD – 1000 USD	254	39.10
1001 USD – 1500 USD	80	12.30
1501 USD – 2000 USD	46	7.09
More than 2000 USD	50	7.70
IFDFW score	41.14	16.93

SD: standard deviation; BMI: body mass index; IFDFW: InCharge Financial Distress/Financial Well-Being Scale.

### Clinical and psychosocial characteristics

Most patients had generalized seizures (71.10%), with tonic-clonic and absence seizures being the most frequent types. Among focal seizures (28.90%), simple partial seizures were the most common. Seizure control was achieved in 67.95% of cases, with patients using an average of 1.61 (± 0.82) antiepileptic drugs. Mean LMAS-14 and LAEP scores were 49.02 (± 8.85) and 42.44 (± 12.47), respectively. The majority of patients (65.33%) did not have their antiepileptic medications covered by public or private insurance and were therefore self-paying for treatment. In addition, nearly half reported obtaining medications from outside Lebanon, often with difficulty. Patients had a mean of 2.16 (± 2.06) comorbidities. Psychosocial assessments showed mean scores of 0.93 (± 1.20) on the ESS, 25.92 (± 18.59) on ABNAS, and 17.67 (± 8.90) on LAS-10. The QOLIE-31 had a mean score of 55.47 (± 17.69). Detailed clinical and psychosocial characteristics are presented in Table 2.

**Table 2:** Clinical and psychosocial characteristics of the study sample.

Variable	Mean or frequency	SD or %
Seizure characteristics		
Focal	183	28.90
Generalized	451	71.10
Type of seizure		
Tonic-clonic	123	19.00
Tonic	69	10.60
Simple partial	113	17.40
Secondarily generalized	29	4.47
Myoclonic	38	5.86
Complex partial	70	10.80
Clonic	25	3.85
Atypical absence	29	4.47
Atonic	48	7.40
Absence	90	13.90
Unknown type	15	2.31
Level of seizure control		
Controlled (no seizures in the last 12 months)	441	67.95
Uncontrolled (persistence of seizures despite treatment)	208	32.05
Number of antiepileptic drugs used	1.61	0.82
LAEP score	42.44	12.47
LMAS-14 score	49.02	8.85
Medications covered by any public or private insurance provider		
No	424	65.33
Yes, partially	157	24.19
Yes, fully	68	10.48
Difficulty to obtain medications given the current situation in Lebanon		
No	207	31.90
Yes, some difficulty	227	34.98
Yes, moderate difficulty	147	22.65
Yes, severe difficulty	68	10.48
Have to obtain medications from outside Lebanon		
No	367	56.55
Yes, sometimes	204	31.43
Yes, most of the time	56	8.63
Yes, always	22	3.39
Total number of comorbidities	2.16	2.06
Number of routine or daily medications other than antiepileptic drugs	1.28	2.09
ESS score	0.93	1.20
ABNAS score	25.92	18.59
Taking any medications for nervous system (other than the antiepileptic drug(s)		
No	498	76.73
Yes	151	23.27
LAS-10 score	17.67	8.90
QOLIE-31 score	55.47	17.69

SD: standard deviation; LAEP: Liverpool Adverse Events Profile; LMAS-14: Lebanese Medication Adherence Scale; ESS: Epilepsy Stigma Scale; ABNAS: A–B Neuropsychological Assessment Schedule; LAS-10: Lebanese Anxiety Scale; QOLIE: Quality of Life in Epilepsy.

### Validation of the QOLIE-15

#### Exploratory factor analysis

An EFA was performed on Sample 1 (*N* = 324) to evaluate the latent structure of the QOLIE-15. All 15 items were retained and analyzed using Promax rotation. None of the items demonstrated excessive intercorrelation (*r* > 0.90), weak loadings (< 0.30), or low communalities (< 0.30). Sampling adequacy was confirmed by a KMO value of 0.875, and Bartlett’s test of sphericity was significant (*P* < 0.001), supporting the suitability of the data for factor analysis.

The analysis produced a 5-factor model with eigenvalues greater than 1, explaining 71.68% of the total variance. Factor 1 (cognitive) comprised five items, Factor 2 (psychological) included three items, Factor 3 (therapeutic) consisted of two items, Factor 4 (seizure worry) contained three items, and Factor 5 (social) included two items. The Promax rotated factor solution is displayed in Table 3.

**Table 3:** Promax rotated factor solution of the QOLIE-15 in Sample 1.

QOLIE-15 item #	QOLIE-15 items	Factor 1	Factor 2	Factor 3	Factor 4	Factor 5	h^2^
QOLIE10	I forget things, for example an appointment or where I put an object.	0.925					0.782
QOLIE12	I get confused and forget what I was doing.	0.856					0.714
QOLIE9	I have difficulties remembering names of people.	0.842					0.645
QOLIE11	I have difficulties concentrating on the things I am doing.	0.817					0.755
QOLIE8	My mind does not work as fast as it should.	0.587					0.662
QOLIE13	Physical condition (muscular): Aches and pains, tingling, stiffness, rapid muscle contractions, unsteady voice, increased muscle volume.		0.852				0.711
QOLIE15	Depressed mood: Loss of interest, lack of enjoyment in hobbies, depression, waking up early, mood swings during the day.		0.839				0.790
QOLIE14	Anxious mood: Worries, expecting the worst, a prior feeling of fear, excessive irritability.		0.815				0.775
QOLIE6	Physical aspects of antiepileptic medication.			0.936			0.835
QOLIE7	Mental aspects of antiepileptic medication.			0.894			0.837
QOLIE4	Do you worry about hurting yourself during a seizure?				0.780		0.630
QOLIE5	How worried are you about embarrassment or other social problems resulting from having a seizure during the next month?				0.696		0.607
QOLIE1	Have you worried about having another seizure?				0.673		0.559
QOLIE3	Trouble with driving.					0.872	0.735
QOLIE2	Trouble with leisure time (such as hobbies, going out).					0.835	0.713
	*Percentage of variance explained*	39.56%	8.74%	8.40%	7.93%	7.04%	

Factor 1 = Cognitive; Factor 2 = Psychological; Factor 3 = Therapeutic; Factor 4 = Seizure worry; Factor 5 = Social. h^2^: communalities. QOLIE-15: 15-item Quality of Life in Epilepsy Scale.

Total percentage of variance explained: 71.68%.

Kaiser-Meyer-Olkin (KMO) = 0.875.

Bartlett’s test of sphericity: *P* < 0.001.

#### Confirmatory factor analysis

**Fig. 1:**
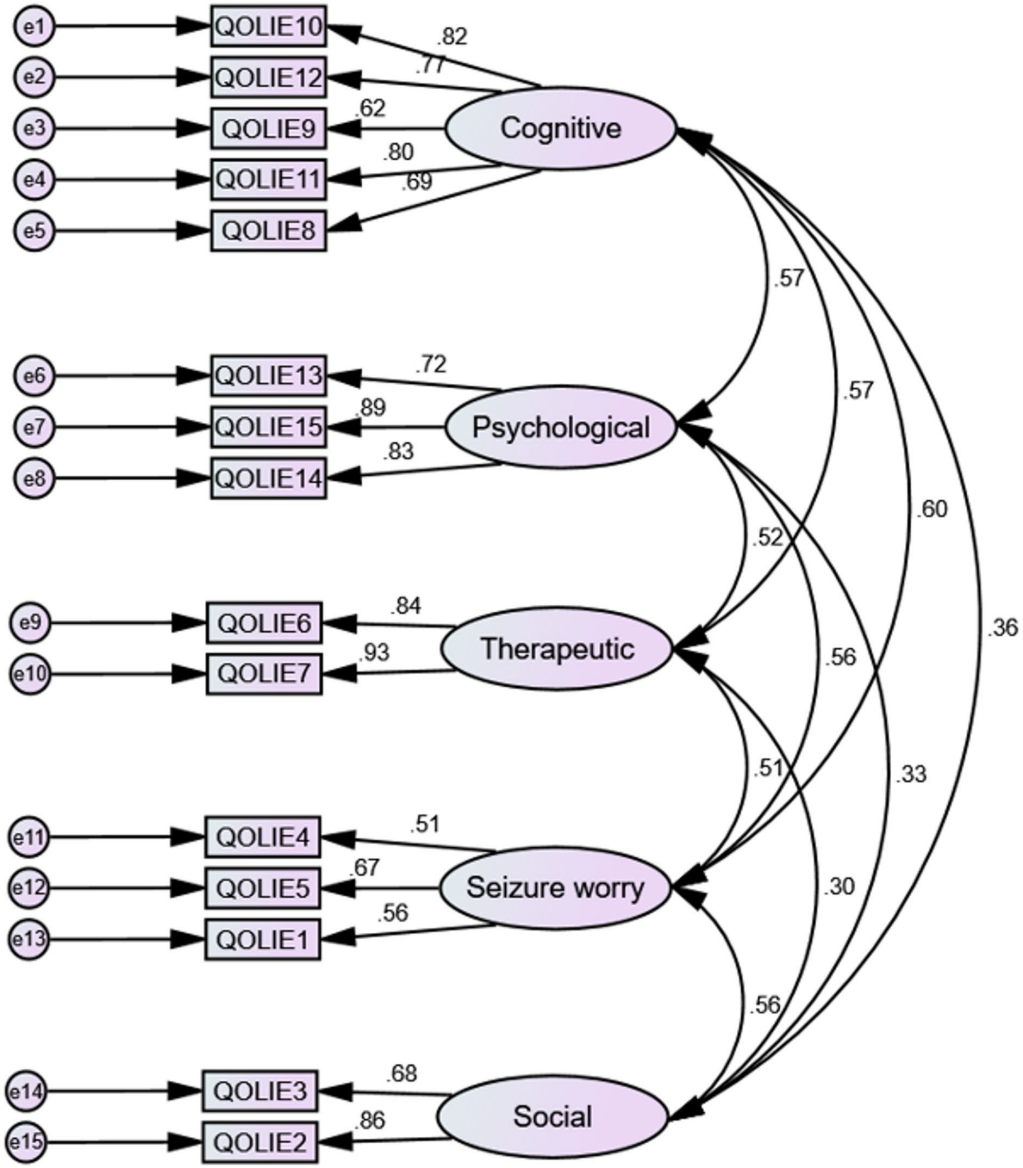
Standardized estimates of factor loadings from the confirmatory factor analysis (CFA) of the QOLIE-15 items in Sample 2. QOLIE: Quality of Life in Epilepsy. e = error term.

A CFA was performed on Sample 2 (*N* = 350) to test the 5-factor solution of the QOLIE-15 derived from the EFA in Sample 1. Using maximum likelihood estimation, the model demonstrated a good overall fit, with χ^2^/df = 124.598/80 = 1.557 (*P* = 0.001). Additional fit indices further supported the adequacy of the model. The CFI (0.978) and TLI (0.972) both surpassed the recommended cutoff of 0.95. The RMSEA was 0.041 (90% CI: 0.024–0.055), with a close-fit test yielding a nonsignificant result (*P* = 0.838), confirming excellent fit. The SRMR was 0.034, well below the accepted threshold of 0.08. Figure 1 presents the standardized factor loadings and structural paths for the QOLIE-15 CFA model.

#### Measurement invariance

Multigroup CFA in the total sample supported configural, metric, and scalar invariance of the 5-factor QOLIE-15 model across gender, seizure characteristics, and seizure control. In all models, the fit indices demonstrated good model fit (CFI values ≥ 0.973, RMSEA ≤ 0.046, SRMR ≤ 0.043). Comparisons between successive models showed negligible differences in fit, with ΔCFI ≤ 0.001, ΔRMSEA ≤ 0.002, and ΔSRMR ≤ 0.005. These results confirm that the factorial structure of the QOLIE-15 was consistent across the examined subgroups. Detailed multigroup invariance results are presented in Table 4.

**Table 4:** Multigroup CFA of QOLIE-15 measurement invariance across gender, seizure characteristics, and seizure control.

Model	CFI	RMSEA	SRMR	Model comparison	ΔCFI	ΔRMSEA	ΔSRMR
Model 1: across gender (male vs. female)							
Configural	0.974	0.046	0.037				
Metric	0.974	0.045	0.042	Configural vs. metric	< 0.001	0.001	0.005
Scalar	0.973	0.044	0.043	Metric vs. scalar	0.001	0.001	0.001
Model 2: across seizure characteristics (generalized vs. focal)							
Configural	0.975	0.045	0.036				
Metric	0.974	0.044	0.040	Configural vs. metric	0.001	0.001	0.004
Scalar	0.975	0.042	0.040	Metric vs. scalar	0.001	0.002	< 0.001
Model 3: across seizure control (controlled vs. uncontrolled)							
Configural	0.974	0.044	0.037				
Metric	0.973	0.044	0.041	Configural vs. metric	0.001	< 0.001	0.004
Scalar	0.973	0.042	0.042	Metric vs. scalar	< 0.001	0.002	0.001

CFI, comparative fit index; RMSEA, root mean square error of approximation; SRMR, standardized root mean square residual.

#### Internal consistency and reliability

**Fig. 2:**
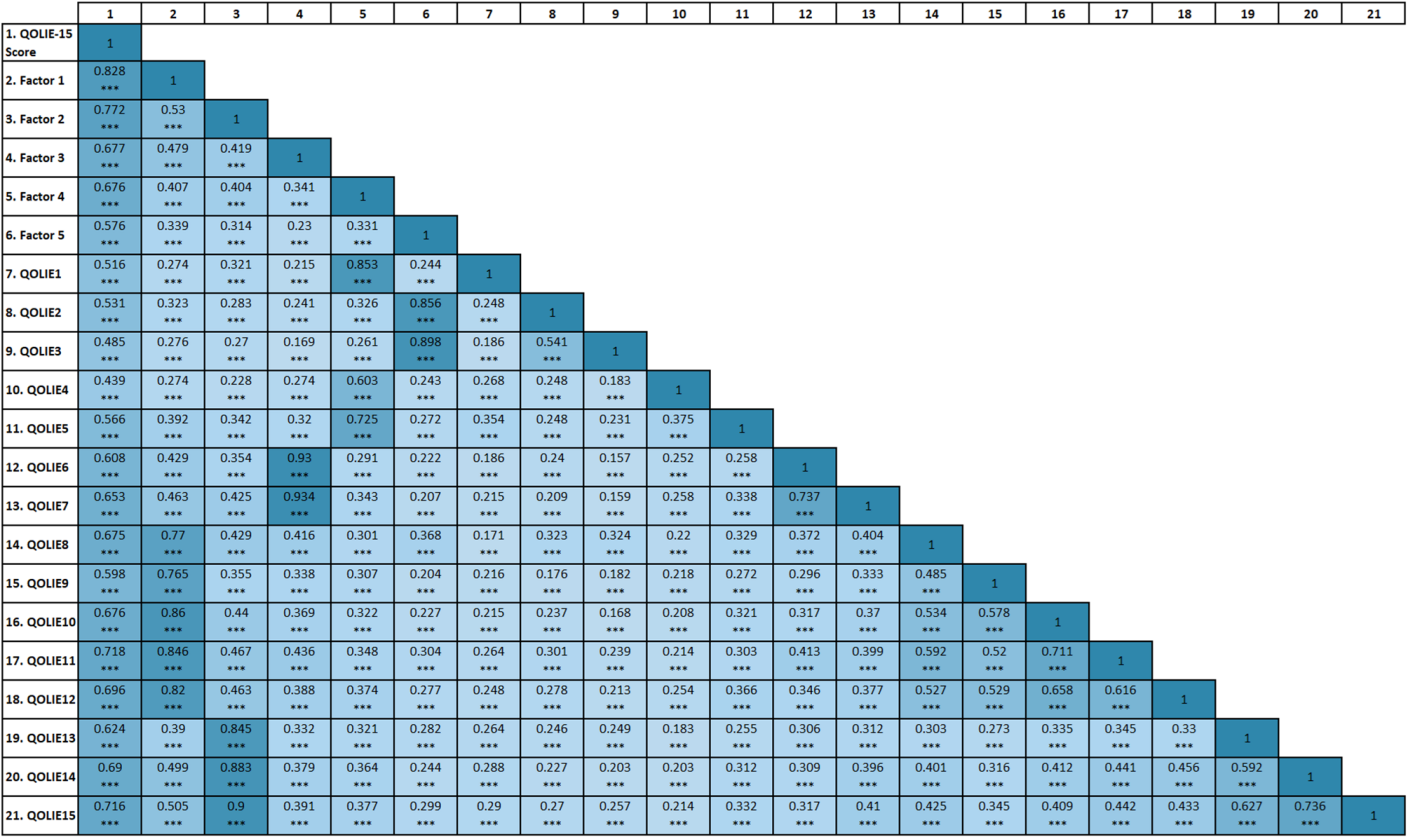
Pearson correlation matrix for the QOLIE-15 items, subscales, and total score. QOLIE-15: 15-item Quality of Life in Epilepsy Scale; Factor 1 (Cognitive); Factor 2 (Psychological); Factor 3 (Therapeutic); Factor 4 (Seizure worry); Factor 5 (Social); QOLIE1 to QOLIE15 (QOLIE-15 scale item numbers). ^***^P < 0.001.

Figure 2 displays the Pearson correlation matrix for the QOLIE-15 total score, its five factor subscales, and individual items in the total sample. All coefficients were statistically significant at *P* < 0.001. The subscales were strongly correlated with the total score (Factor 1: *r* = 0.828; Factor 2: *r* = 0.772; Factor 3: *r* = 0.677; Factor 4: *r* = 0.676; Factor 5: *r* = 0.576), underscoring the multidimensional yet coherent structure of the QOLIE-15. Within each subscale, items demonstrated moderate to very high correlations with their respective factor, further confirming the internal consistency of the scale.

Table 5 presents the internal consistency reliability estimates for the QOLIE-15. The total scale demonstrated excellent reliability (α = 0.906; ω = 0.929). Subscale reliabilities were also generally high, with α/ω values of 0.907/0.927 for Factor 1, 0.882/0.887 for Factor 2, and 0.890 for Factor 3. Lower values were observed for Factor 4 (α = 0.663; ω = 0.678) and Factor 5 (α = 0.786), though these remained within acceptable ranges for short subscales.

**Table 5:** Internal consistency reliability estimates of the QOLIE-15 scale and subscales.

Scale/subscale	Cronbach’s α	McDonald’s ω
QOLIE-15	0.906	0.929
Factor 1: Cognitive	0.907	0.927
Factor 2: Psychological	0.882	0.887
Factor 3: Therapeutic	0.890	NA
Factor 4: Seizure worry	0.663	0.678
Factor 5: Social	0.786	NA

QOLIE-15: 15-item Quality of Life in Epilepsy Scale; NA = not applicable (McDonald’s ω not computed for factors with fewer than 3 items).

#### Convergent and concurrent

Evidence for convergent validity was provided by the strong positive correlations of the QOLIE-15 with the QOLIE-31 (*r* = 0.875, *P* < 0.001). Concurrent validity was further supported by significant correlations between the QOLIE-15 and related measures, including the LMAS-14 (*r* = 0.369, *P* < 0.001), LAEP (*r* = -0.650, *P* < 0.001), ABNAS (*r* = -0.830, *P* < 0.001), ESS (*r* = -0.471, *P* < 0.001), and LAS-10 (*r* = -0.814, *P* < 0.001).

#### Criterion validity

**Fig. 3:**
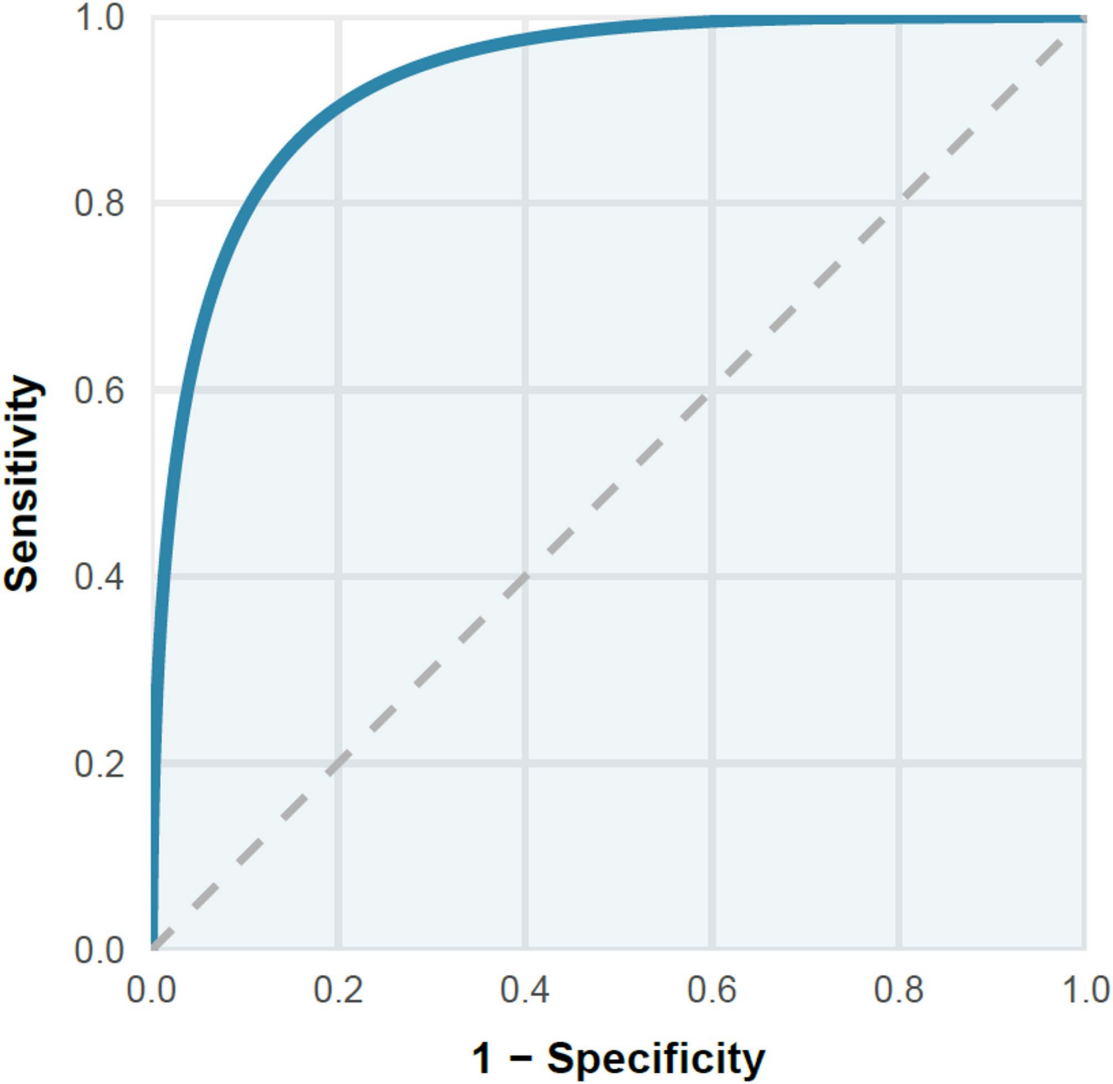
ROC curve of the QOLIE-15 using the QOLIE-31 to classify participants with better quality of life. Area under the curve = 0.936 (95% CI: 0.918–0.953; *P* < 0.001). At the cut-off score of 44.5, sensitivity = 88.80% and specificity = 82.00%.

The mean QOLIE-15 score was 45.91 (± 11.28), where higher values reflect better QOL. To evaluate criterion validity, a ROC curve analysis was conducted using the QOLIE-31 as the reference standard to distinguish between participants with higher versus lower QOL. The analysis indicated an optimal QOLIE-15 threshold of 44.50, which achieved a sensitivity of 88.80% and a specificity of 82.00%. The area under the curve was 0.936 (95% CI: 0.918–0.953; *P* < 0.001), demonstrating excellent discriminative ability. The ROC curve is presented in Fig. 3.

### Multivariable analysis

Table 6 presents three multivariable linear regression models to identify predictors of QOL as measured by the QOLIE-15. Model 1 showed that higher age (B = -0.064), greater House Crowding Index (B = -2.220), and current alcohol use (B = -5.744) were associated with lower QOLIE-15 scores. In contrast, being retired (B = 6.676), reporting easy access to healthcare (B = 3.439), and higher financial wellbeing (IFDFW; B = 0.117) predicted better QOL.

Model 2 showed that uncontrolled seizures (B = -1.543) and higher LAEP scores (B = -0.097) were linked to lower QOL. Psychosocial variables including higher stigma (ESS; B = -0.389), cognitive complaints (ABNAS; B = -0.275), and anxiety (LAS-10; B = -0.520) were also negatively associated with QOL. Conversely, full medication coverage (B = 1.259) was associated with better QOL.

Model 3 showed that greater House Crowding Index (B = -0.773), uncontrolled seizures (B = -1.528), higher LAEP scores (B = -0.097), higher stigma (ESS; B = -0.380), cognitive complaints (ABNAS; B = -0.276), and anxiety (LAS-10; B = -0.524) remained significant negative predictors of QOL.

**Table 6:** Multivariable linear regression of predictors of quality of life (QOLIE-15) in patients with epilepsy.

Variable	Unstandardized Beta (B)	Standardized Beta (β)	95% CI		*P* value
			Lower	Upper	
Model 1: including sociodemographic and socioeconomic characteristics^*^					
Age	− 0.064	− 0.087	− 0.121	− 0.007	0.029
House Crowding Index	− 2.220	− 0.106	− 3.816	− 0.625	0.006
Occupation (Reference: Employed / Self-employed)					
Unemployed	− 0.892	− 0.040	− 2.591	0.807	0.303
Retired	6.676	0.107	1.839	11.513	0.007
Alcohol consumption (Reference: No)					
In the past, not anymore	0.113	0.002	− 3.915	4.141	0.956
Yes, currently	− 5.744	− 0.100	− 10.175	− 1.314	0.011
Easy access to healthcare					
Yes vs. No	3.439	0.128	1.372	5.506	0.001
IFDFW score	0.117	0.175	0.056	0.177	< 0.001
Model 2: including clinical and psychosocial characteristics^**^					
Level of seizure control					
Uncontrolled vs. controlled	− 1.543	− 0.064	− 2.384	− 0.703	< 0.001
LAEP score	− 0.097	− 0.107	− 0.136	− 0.057	< 0.001
Medications covered by any public or private insurance provider (Reference: No)					
Yes, partially	0.659	0.025	− 0.224	1.542	0.143
Yes, fully	1.259	0.034	0.029	2.490	0.045
ESS score	− 0.389	− 0.041	− 0.742	− 0.036	0.031
ABNAS score	− 0.275	− 0.454	− 0.305	− 0.246	< 0.001
LAS-10 score	− 0.520	− 0.410	− 0.579	− 0.460	< 0.001
Model 3: including statistically significant sociodemographic, socioeconomic, clinical, and psychosocial variables from Model 1 and Model 2^***^					
House Crowding Index	− 0.773	− 0.037	− 1.476	− 0.07	0.031
Level of seizure control					
Uncontrolled vs. controlled	− 1.528	− 0.063	− 2.372	− 0.684	< 0.001
LAEP score	− 0.097	− 0.107	− 0.136	− 0.058	< 0.001
Medications covered by any public or private insurance provider (Reference: No)					
Yes, partially	0.571	0.022	− 0.313	1.456	0.205
Yes, fully	1.225	0.033	− 0.006	2.456	0.051
ESS score	− 0.380	− 0.041	− 0.735	− 0.026	0.035
ABNAS score	− 0.276	− 0.454	− 0.305	− 0.246	< 0.001
LAS-10 score	− 0.524	− 0.413	− 0.583	− 0.464	< 0.001

95% CI: 95% confidence interval; USD: US dollars; IFDFW: InCharge Financial Distress/Financial Well-Being Scale; LAEP: Liverpool Adverse Events Profile; ESS: Epilepsy Stigma Scale; ABNAS: A-B Neuropsychological Assessment Schedule; LAS-10: Lebanese Anxiety Scale.

^*^Variables initially included in the model: age; House Crowding Index; area of residence; marital status; level of education; occupation; smoking status; alcohol consumption; easy access to healthcare; household monthly income; IFDFW (InCharge Financial Distress/Financial Well-Being Scale) score.

^**^Variables initially included in the model: type of seizure; level of seizure control; number of antiepileptic drugs used; LAEP (Liverpool Adverse Events Profile) score; LMAS-14 (Lebanese Medication Adherence Scale) score; medications coverage by any public or private insurance provider; difficulty to obtain medications given the current situation in Lebanon; have to obtain medications from outside Lebanon; total number of comorbidities; number of routine or daily medications other than antiepileptic drugs; ESS (Epilepsy Stigma Scale) score; ABNAS (A-B Neuropsychological Assessment Schedule) score; taking any medications for nervous system other than the antiepileptic drug; LAS-10 (Lebanese Anxiety Scale) score.

^***^Variables initially included in the model: age; House Crowding Index; occupation; alcohol consumption; easy access to healthcare; household monthly income; IFDFW (InCharge Financial Distress/Financial Well-Being Scale) score; level of seizure control; LAEP (Liverpool Adverse Events Profile) score; medications coverage by any public or private insurance provider; ESS (Epilepsy Stigma Scale) score; ABNAS (A-B Neuropsychological Assessment Schedule) score; LAS-10 (Lebanese Anxiety Scale) score.

## Discussion

This study introduced and validated the QOLIE-15 as a concise, multidimensional instrument for assessing QOL in epilepsy. While informed by content from existing validated instruments, the QOLIE-15 was developed and validated as an independent measure with its own factorial structure and scoring framework. The scale demonstrated a stable five-factor structure, strong internal consistency, and measurement invariance across epilepsy subgroups. It showed excellent psychometric performance, convergent, concurrent, and criterion validity, with strong discriminatory capacity. Multivariable analyses identified several determinants of QOL. Consistent with contemporary analytical recommendations, the regression models were theory- and practice-driven. Covariates were selected a priori based on clinical and contextual relevance to epilepsy-related QOL, which enhances interpretability and generalizability of the findings. Among sociodemographic and socioeconomic factors, older age, greater household crowding, and alcohol use were associated with poorer outcomes, whereas retirement, better healthcare access, financial wellbeing, and medication coverage were linked to higher QOL. Clinically, uncontrolled seizures and treatment-related adverse effects, and psychosocially, cognitive complaints, anxiety, and stigma, were significant negative correlates. It is important to note that seizure control in this cohort does not equate to absence of clinical complexity, as all participants were receiving antiepileptic treatment and many continued to experience adverse effects, cognitive complaints, and psychosocial challenges. Thus, the proportion of controlled patients should not be interpreted as reflecting a less severe epilepsy population.

The validation process confirmed the theoretical multidimensionality of epilepsy-related QOL. EFA revealed five coherent domains: cognition, psychological wellbeing, therapeutic adverse effects, seizure worry, and social functioning, aligned with the conceptual model underlying QOLIE-31^36^. CFA showed robust fit indices that exceeded recommended thresholds^31,32^. In contrast to reports of inconsistent structures for QOLIE-31^15,37^, and QOLIE-10^16,17,38^ across cultures, and given the lack of evidence on their model fits, the replicable structure of QOLIE-15 is a key strength. Multigroup CFA established invariance across gender, seizure characteristics, and seizure control, supporting fair subgroup comparisons. To our knowledge, such invariance testing has not been reported for QOLIE-31 or QOLIE-10, positioning QOLIE-15 as a more rigorously validated alternative.

Reliability analyses further supported the QOLIE-15, with excellent internal consistency for the total scale, comparable or superior to QOLIE-31 and QOLIE-10^38–41^. Subscale reliabilities were similarly high, except for seizure worry, which is acceptable given its brevity and conceptual specificity^30^. Construct validity was strong: convergent validity via a high correlation with QOLIE-31, and concurrent validity through expected associations with adverse events (LAEP), cognitive complaints (ABNAS), anxiety (LAS-10), and stigma (ESS), consistent with broader evidence linking these factors to poorer QOL^42–45^. Criterion validity was excellent, with high sensitivity and specificity in distinguishing higher versus lower QOL when QOLIE-31 was used as the reference, consistent with prior evidence supporting the clinical utility of epilepsy-specific measures^11,12^.

Regarding predictors, lower QOL was associated with older age, household crowding, and alcohol use, while retirement, healthcare access, and financial wellbeing related to higher QOL. The age gradient aligns with reports that advancing age and longer disease duration impair multiple QOL domains and are associated with poorer HRQoL^46^. Likely mechanisms include accumulating comorbidities, polypharmacy, and functional limitations^47^. The association between household crowding, as a marker of social deprivation, and lower QOL is consistent with findings that structural hardship restricts service access and self-management^48^. Alcohol use also predicted worse QOL, consistent with literature linking alcohol to seizure risk and exacerbations^49,50^.

Conversely, better healthcare access and financial wellbeing were linked to higher QOL, in line with evidence that cost-related barriers reduce specialist use and worsen outcomes^51^. A systematic review also highlighted socioeconomic status as a notable determinant of HRQoL in patients with epilepsy^52^. However, retirement was associated with better QOL, which may reflect reduced seizure triggers and job-related stressors, consistent with prior observations that psychosocial load can outweigh the generic employment advantage^53^.

Among clinical predictors, uncontrolled seizures, greater adverse-effect burden, stigma, cognitive complaints, anxiety, and lack of full medication coverage independently reduced QOL. The current impact of seizure control reproduces longstanding evidence that seizure freedom drives HRQoL gains^48,54^. Adverse events also depressed QOL, echoing work showing that LAEP scores predict lower wellbeing and mood, and consistent with reports that antiepileptic drug side effects impair energy, cognition, and mood^55–57^. Full medication coverage, by contrast, was associated with better QOL, aligning with evidence that coverage gaps constrain epilepsy care^51^, and with economic determinants of HRQoL summarized in prior reviews^52^.

Psychosocial factors showed strong independent effects. Stigma was linked to lower QOL, consistent with European survey data and subsequent reviews identifying stigmatization as a major determinant of HRQoL^26,48,58^. Cognitive complaints on ABNAS showed a large negative association. ABNAS captures patient-perceived cognitive side effects related to neuropsychological performance and daily function^27^, and cognitive impairment is a recognized driver of HRQoL decrements^47^. Anxiety was likewise robustly associated with lower QOL, paralleling evidence of poorer QOL during usual care when anxiety or depression are present^52,59^.

Although medication adherence was theoretically expected to correlate with QOL, it did not remain an independent predictor after adjustment for clinical and psychosocial variables. Two explanations fit the literature. First, adherence likely influences QOL indirectly via seizure control and emotional load^52,60^. Once seizures, anxiety, and stigma are modeled, the direct adherence-QOL path attenuates. Second, greater adherence can increase exposure to dose-related adverse effects, which exert strong, independent negative effects on HRQoL. Thus, in patients on polytherapy or higher doses, tolerability may offset QOL gains from improved control^55,57^. Future mediation analyses are suggested to test these pathways. Notably, the persistence of seizure control, adverse events, stigma, cognition, and anxiety in Model 3 underscores their additive and distinct contributions, consistent with prior multivariable work showing that tolerability, affective symptoms, seizure worry, and stigmatization independently shape HRQoL^48^.

### Practical implications

The QOLIE-15 provides clinicians and researchers with a brief yet comprehensive tool to evaluate epilepsy-specific QOL. Its straightforward scoring makes it suitable for clinical encounters, while its multidimensionality allows for targeted identification of modifiable problems, such as anxiety, stigma, or treatment side effects, beyond seizure control. In research, its measurement invariance across key subgroups ensures fair comparisons, supporting use in both trials and population-based studies. Similar to other instruments initially developed and validated in Lebanon and therefore labeled as Lebanese, the QOLIE-15 is also referred to as the Lebanese HRQoL Assessment Scale in Epilepsy (QOLIE-L). In addition, this study offers context-specific insights that can guide individualized patient care. Identification of sociodemographic, socioeconomic, clinical, and psychosocial determinants informs resource allocation and healthcare planning, emphasizing interventions that address both biomedical and social needs.

### Strengths and limitations

This study has several strengths. The QOLIE-15 was developed through a rigorous methodology, ensuring content validity and cultural appropriateness. Psychometric testing included both EFA and CFA, strengthening confidence in the stability of the measurement model. Formal measurement invariance testing across subgroups, lacking in QOLIE-31 or QOLIE-10 validation studies, enhances generalizability. Moreover, the simultaneous examination of diverse clinical and psychosocial predictors offers a multidimensional perspective. The relatively large and diverse sample further supports representativeness.

Nevertheless, several limitations should be acknowledged. The cross-sectional design precludes establishing temporal or causal relationships between predictors and QOL, limiting conclusions to associations rather than directionality. Reliance on self-reported data for certain variables, such as medication adherence or alcohol consumption, may have introduced recall or social desirability bias, potentially leading to under- or overestimation of true associations. The absence of test-retest reliability assessment prevents conclusions about the instrument’s stability over time. Lastly, although variables were prespecified to minimize data-driven bias, residual confounding may still have influenced the observed associations. Future research is warranted to address these limitations.

## Conclusion

The QOLIE-15 is a valid, reliable, and practical instrument that balances brevity with conceptual richness. It retains the multidimensional framework necessary to capture the lived experience of epilepsy, with coherent dimensions that encompass cognitive, psychological, social, therapeutic, and seizure-worry domains, while reducing burden compared with existing measures. The scale demonstrated excellent psychometric properties and identified seizure control, adverse effects, cognition, anxiety, and stigma as independent determinants of QOL. Its simplicity and rigor make it a valuable tool for clinical care, research, and health policy in epilepsy.

## Supplementary Information

Below is the link to the electronic supplementary material.

Supplementary Material 1

Supplementary Material 2

Supplementary Material 3

## Data Availability

The datasets generated during and/or analyzed during the current study are available from the corresponding author upon reasonable request.
