# Supplementary material for: Development, psychometric validation, and correlates of the 15-item quality of life in epilepsy scale (QOLIE-15)
Source: Sci Rep. 2026 Mar 29;16:10678. doi: 10.1038/s41598-026-46379-z (PMC13039895; doi:10.1038/s41598-026-46379-z)
Supplement: Supplementary file 1 — Supplementary Material 1 [file 41598_2026_46379_MOESM1_ESM.pdf]

QOLIE Version 1 (28 Items)

Item #	Matching Scale	Matching Scale Item #	Item
1	LMAS-14	3	Do you forget to take your medication?
2	LMAS-14	12	Do you stop taking your medication in case of side effects?
3	QOLIE-31	6	Did you have a lot of energy?
4	QOLIE-31	9	Have you been a happy person?
5	QOLIE-31	11	Have you worried about having another seizure?
6	QOLIE-31	13	Has your health limited your social activities (such as visiting with friends or close relatives)?
7	QOLIE-31	14	How has the QUALITY OF YOUR LIFE been during the past 4 weeks (that is, how have things been going for you)?
8	QOLIE-31	16	Trouble remembering things people tell you.
9	QOLIE-31	17	Trouble concentrating or reading.
10	QOLIE-31	18	Trouble concentrating or doing one thing at a time.
11	QOLIE-31	19	The following questions are about problems you may have with certain ACTIVITIES. Choose one number for how much during the past 4 weeks your epilepsy or antiepileptic medication has caused trouble with…Leisure.
12	QOLIE-31	20	The following questions are about problems you may have with certain ACTIVITIES. Choose one number for how much during the past 4 weeks your epilepsy or antiepileptic medication has caused trouble with…Driving.
13	QOLIE-31	21	How fearful are you of having a seizure during the next month?
14	QOLIE-31	22	Do you worry about hurting yourself during a seizure?
15	QOLIE-31	23	How worried are you about embarrassment or other social problems resulting from having a seizure during the next month?
16	QOLIE-31	29	For each of these PROBLEMS, choose one number for how much they bother you on a scale of what 1 to 5 where 1= Extremely bothersome, and 5= Not at all bothersome.<br>Physical aspects of antiepileptic medication.
17	QOLIE-31	30	For each of these PROBLEMS, choose one number for how much they bother you on a scale of what 1 to 5 where 1= Extremely bothersome, and 5= Not at all bothersome.<br>Mental aspects of antiepileptic medication.
18	ABNAS	2	My mind does not work as fast as it should.
19	ABNAS	3	I have difficulties remembering names of people.
20	ABNAS	9	I forget things, for example an appointment or where I put an object.

21	ABNAS	10	I have difficulties concentrating on the things I am doing.
22	ABNAS	16	I can't concentrate for more than a short period of time.
23	ABNAS	20	I get confused and forget what I was doing.
24	LAS-10	1	Insomnia: Difficulty falling asleep, interrupted sleep, unsatisfactory sleep duration, feeling tired upon waking up, dreams, nightmares, night terrors.
25	LAS-10	2	Tension: Feelings of stress, fatigue, startling easily, crying easily, trembling, feelings of restlessness, and inability to relax.
26	LAS-10	3	Physical condition (muscular): Aches and pains, tingling, stiffness, rapid muscle contractions, unsteady voice, increased muscle volume.
27	LAS-10	4	Anxious mood: Worries, expecting the worst, a prior feeling of fear, excessive irritability.
28	LAS-10	5	Depressed mood: Loss of interest, lack of enjoyment in hobbies, depression, waking up early, mood swings during the day.
