# Supplementary material for: Development, psychometric validation, and correlates of the 15-item quality of life in epilepsy scale (QOLIE-15)
Source: Sci Rep. 2026 Mar 29;16:10678. doi: 10.1038/s41598-026-46379-z (PMC13039895; doi:10.1038/s41598-026-46379-z)
Supplement: Supplementary file 2 — Supplementary Material 2 [file 41598_2026_46379_MOESM2_ESM.pdf]

QOLIE Version 2 (18 Items)

Item #	Matching Scale	Matching Scale Item #	Item
1	QOLIE-31	6	Did you have a lot of energy?
2	QOLIE-31	11	Have you worried about having another seizure?
3	QOLIE-31	13	Has your health limited your social activities (such as visiting with friends or close relatives)?
4	QOLIE-31	16	Trouble remembering things people tell you.
5	QOLIE-31	19	The following questions are about problems you may have with certain ACTIVITIES. Choose one number for how much during the past 4 weeks your epilepsy or antiepileptic medication has caused trouble with…Leisure.
6	QOLIE-31	20	The following questions are about problems you may have with certain ACTIVITIES. Choose one number for how much during the past 4 weeks your epilepsy or antiepileptic medication has caused trouble with…Driving.
7	QOLIE-31	22	Do you worry about hurting yourself during a seizure?
8	QOLIE-31	23	How worried are you about embarrassment or other social problems resulting from having a seizure during the next month?
9	QOLIE-31	29	For each of these PROBLEMS, choose one number for how much they bother you on a scale of what 1 to 5 where 1= Extremely bothersome, and 5= Not at all bothersome. Physical aspects of antiepileptic medication.
10	QOLIE-31	30	For each of these PROBLEMS, choose one number for how much they bother you on a scale of what 1 to 5 where 1= Extremely bothersome, and 5= Not at all bothersome. Mental aspects of antiepileptic medication.
11	ABNAS	2	My mind does not work as fast as it should.
12	ABNAS	3	I have difficulties remembering names of people.
13	ABNAS	9	I forget things, for example an appointment or where I put an object.
14	ABNAS	10	I have difficulties concentrating on the things I am doing.
15	ABNAS	20	I get confused and forget what I was doing.
16	LAS-10	3	Physical condition (muscular): Aches and pains, tingling, stiffness, rapid muscle contractions, unsteady voice, increased muscle volume.
17	LAS-10	4	Anxious mood: Worries, expecting the worst, a prior feeling of fear, excessive irritability.

18	LAS-10	5	Depressed mood: Loss of interest, lack of enjoyment in hobbies, depression, waking up early, mood swings during the day.
