# Supplementary material for: Development, psychometric validation, and correlates of the 15-item quality of life in epilepsy scale (QOLIE-15)
Source: Sci Rep. 2026 Mar 29;16:10678. doi: 10.1038/s41598-026-46379-z (PMC13039895; doi:10.1038/s41598-026-46379-z)
Supplement: Supplementary file 3 — Supplementary Material 3 [file 41598_2026_46379_MOESM3_ESM.pdf]

QOLIE-15 Final Version

English Version

This question is about how you FEEL and how things have been for you during the past 4 weeks. Please indicate the one answer that comes closest to the way you have been feeling.

1. Have you worried about having another seizure?	All of the time 1 ☐	Most of the time 2 ☐	A good bit of the time 3 ☐	Some of the time 4 ☐	A little of the time 5 ☐	None of the time 6 ☐

The following questions are about problems you may have with certain ACTIVITIES. Choose one number for how much during the past 4 weeks your epilepsy or antiepileptic medication has caused trouble with…

	A great deal	A lot	Somewhat	Only a little	Not at all
2. Leisure time (such as hobbies, going out)	1 ☐	2☐	3 ☐	4 ☐	5 ☐
3. Driving	1 ☐	2☐	3 ☐	4 ☐	5 ☐

4. Do you worry about hurting yourself during a seizure?

- ☐ (1) Worry a lot
- ☐ (2) Occasionally worry
- ☐ (3) Don’t worry at all

5. How worried are you about embarrassment or other social problems resulting from having a seizure during the next month?	Very worried 1 ☐	Somewhat worried 2☐	Not very worried 3 ☐	Not at all worried 4 ☐

For each of these PROBLEMS, choose one number for how much they bother you on a scale of what 1 to 5 where 1= Extremely bothersome, and 5= Not at all bothersome.

	Extremely bothersome				Not at all bothersome
6. Physical aspects of antiepileptic medication	1 ☐	2☐	3 ☐	4 ☐	5 ☐

7. Mental aspects of antiepileptic medication	1 ☐	2☐	3 ☐	4 ☐	5 ☐

Below is a list of problems people sometimes have with epilepsy or the medicine they take for their epilepsy. Have you had any problems listed which you think may have been caused by epilepsy or the antiepileptic drug(s) you take? For each item, if it is not a problem choose 4; if it is a mild problem choose 3; if it is a moderate problem choose 2; and if it is a serious problem choose 1.

	**No problem**	**A mild problem**	**A moderate problem**	**A serious problem**
8. My mind does not work as fast as it should	4 ☐	3 ☐	2 ☐	1 ☐
9. I have difficulties remembering names of people	4 ☐	3 ☐	2 ☐	1 ☐
10. I forget things, for example an appointment or where I put an object	4 ☐	3 ☐	2 ☐	1 ☐
11. I have difficulties concentrating on the things I am doing	4 ☐	3 ☐	2 ☐	1 ☐
12. I get confused and forget what I was doing	4 ☐	3 ☐	2 ☐	1 ☐

Please choose the answer that best describes your current situation for each of the below statements.

	**None**	**Mild**	**Moderate**	**Severe**	**Very severe**
13. Physical condition (muscular): Aches and pains, tingling, stiffness, rapid muscle contractions, unsteady voice, increased muscle volume.	5 ☐	4 ☐	3 ☐	2 ☐	1 ☐
14. Anxious mood: Worries, expecting the worst, a prior feeling of fear, excessive irritability.	5 ☐	4 ☐	3 ☐	2 ☐	1 ☐
15. Depressed mood: Loss of interest, lack of enjoyment in hobbies, depression, waking up early, mood swings during the day.	5 ☐	4 ☐	3 ☐	2 ☐	1 ☐

Arabic Version

يتعلق هذا السؤال بكيفية **شعورك** وبما مررت به **خلال الأسابيع الأربعة الماضية**. يرجى اختيار الإجابة التي تعبّر بشكل أقرب عن شعورك.

.1 هل كنت قلقًا بشأن حدوث نوبة صرع أخرى؟	طوال الوقت (1)	معظم الوقت (2)	**جزء كبير من الوقت (3)**	**بعض الوقت (4)**	**القليل من الوقت (5)**	**أبدًا (6)**

الأسئلة التالية تتعلق بالمشاكل التي قد تواجهها في بعض **الأنشطة**.

اختر رقمًا واحدًا يعبّر عن **مدى تأثير** مرض الصرع أو الأدوية المضادة للصرع في التسبب بالمشاكل التالية **خلال الأسابيع الأربعة الماضية**...

	بدرجة كبيرة جدًا	بدرجة كبيرة	إلى حد ما	بدرجة بسيطة فقط	على الإطلاق لا
2. وقت الفراغ (مثل الهوايات، الخروج)	1 ☐	2☐	3☐	4☐	5☐
3. القيادة	1 ☐	2☐	3☐	4☐	5☐

4. هل تشعر بالقلق بشأن إيذاء نفسك أثناء النوبة؟

- ☐ (1) أقلق كثيرًا
- ☐ (2) أقلق أحيانًا
- ☐ (3) لا أقلق على الإطلاق

5. ما مدى قلقك من الإحراج أو المشاكل الاجتماعية الأخرى التي قد تنشأ نتيجة لحدوث نوبة صرع خلال الشهر القادم؟	قلق جدًا<br>1 ☐	قلق إلى حد ما<br>2 ☐	لست قلقا جدا<br>3 ☐	لست قلقا على الاطلاق<br>4 ☐

بالنسبة لكل من هذه **المشاكل**، اختر رقمًا واحدًا يعبر عن **مدى إزعاجها لك** على مقياس من 1 إلى 5، حيث 1 = مزعج للغاية، و 5 = غير مزعج على الاطلاق.

	مزعج للغاية				غير مزعج على الاطلاق
6. الجوانب الجسدية للأدوية المضادة للصرع	1 ☐	2☐	3☐	4☐	5☐

7. الجوانب النفسية للأدوية المضادة للصرع	1 ☐	2☐	3☐	4☐	5☐

فيما يلي قائمة بالمشاكل التي قد يواجهها الأشخاص أحيانًا بسبب الصرع أو الأدوية التي يتناولونها لعلاج الصرع. هل واجهت أي مشاكل مدرجة تعتقد أنها قد تكون ناجمة عن الصرع أو الأدوية التي تتناولها؟ لكل عنصر، إذا لم يكن هناك مشكلة اختر 4؛ إذا كانت مشكلة بسيطة اختر 3؛ إذا كانت مشكلة متوسطة اختر 2؛ وإذا كانت مشكلة خطيرة اختر 1.

	لا مشكلة	مشكلة بسيطة	مشكلة متوسطة	مشكلة خطيرة
8. عقلي لا يعمل بالسرعة التي يجب أن يعمل بها	4 ☐	3 ☐	2 ☐	1 ☐
9. أواجه صعوبة في تذكر أسماء الأشخاص	4 ☐	3 ☐	2 ☐	1 ☐
10. أنسى الأشياء، مثل مواعيد أو مكان وضع الأشياء	4 ☐	3 ☐	2 ☐	1 ☐
11. أجد صعوبة في التركيز على الأشياء التي أقوم بها	4 ☐	3 ☐	2 ☐	1 ☐
12. أرتبك وأنسى ما كنت أفعله	4 ☐	3 ☐	2 ☐	1 ☐

الرجاء تحديد الإجابة الصحيحة الى كل من العناصر التالية:

	شديد جدا	شديد	متوسط	معتدل	لا يوجد
13. حالة جسدية (عضلية): الآلام والأوجاع، والوخز، وصلابة، إنكماش سريع في العضلات، صوت متقلب، زيادة حجم العضلات.	1	2	3	4	5
14. مزاج قلق: هموم، توقّع الأسوأ، شعور سابق للخوف، تهيّج مفرط	1	2	3	4	5
15. مزاج مكتئب : فقدان الاهتمام، وعدم المتعة في ممارسة الهوايات، والاكتئاب، والاستيقاظ المبكر، و تقلّبات المزاج خلال النهار	1	2	3	4	5
